# The Role of the 14–20 Domain of the Islet Amyloid Polypeptide in Amyloid Formation

**DOI:** 10.1155/2008/256954

**Published:** 2008-06-10

**Authors:** Sharon Gilead, Ehud Gazit

**Affiliations:** Department of Molecular Microbiology and Biotechnology, George S. Wise Faculty of Life Sciences, Tel Aviv University, Tel Aviv 69978, Israel

## Abstract

The molecular mechanism of amyloid formation by the islet amyloid polypeptide (IAPP) has been intensively studied since its identification in the late 1980s. The IAPP(20–29) region is considered to be the central amyloidogenic module of the polypeptide. This assumption is mainly based on the amyloidogenic properties of the region and on the large sequence diversity within this region between the human and mouse IAPP, as the mouse IAPP does not form amyloids. A few years ago, another region within IAPP was identified that seems to be at least as important as IAPP(20–29) in facilitation of molecular recognition that leads to amyloid formation. Here, we reinforce our and others' previous findings by analyzing supporting evidence from the recent literature. Moreover, we provide new proofs to our hypothesis by comparing between the amyloidogenic properties of the two regions derived from the IAPP of cats, which is also known to form amyloid fibrils.

## 1. INTRODUCTION

The islet amyloid polypeptide (IAPP) is a 37 amino acid hormone, which is colocalized with insulin in the pancreatic *β*-cells. The two polypeptides are cosecreted in response to *β*-cell stimulation [1–4]. In 1986 and 1987, Westermark et al. and Cooper et al. identified IAPP (also designated as *Amylin*) as the major component of pancreatic amyloid deposits, a characteristic pathological feature of type II diabetes [5, 6]. As other amyloid-related proteins and polypeptides, the IAPP identified in these deposits is the wild-type polypeptide in most type II diabetes cases. In its physiological role, IAPP acts as a regulator of glucose homeostasis [7–9].

The formation of amyloid fibrils, which is the hallmark of a group of more than twenty diseases, among them some of the most devastating disorders of the 20th century, is a highly specific self-assembly process [10–14]. The accumulation of IAPP, as well as of amyloidogenic proteins found in other amyloid-related diseases, into amyloid fibrils proceeds via a self-recognition mechanism. The process involves a structural transition from the protein native structure, which is considered to be random coil in the case of IAPP, into a cross-*β*-pleated-sheet secondary structure conformation.

The exact conditions that stimulate the aggregation of amyloidogenic proteins are yet to be fully understood. However, comprehension of the molecular traits of the amyloidogenic process may be highly valuable in the attempts to understand and prevent this world affecting phenomenon. The identification of IAPP in the late 1980s paved the way for vast investigation regarding the molecular mechanism of IAPP self-assembly process. A central issue addressed was the search for the molecular elements that make IAPP highly prone to amyloid formation. One of the first observations regarding IAPP amyloidogenicity was the specie specificity of this process [15, 16]. Although more than 80% of the IAPP sequence is conserved in mammalians, only a few species such as humans, primates, and cats develop islet amyloid and suffer from type II diabetes, while mice, rats, and dogs do not develop islet amyloid deposits.

The most noticeable sequence diversity is the one between human and mouse/rat IAPP. The sequence of rodent IAPP (rIAPP) differs from that of human IAPP (hIAPP) in 6 out of 37 amino acids, 5 of them are located in a defined region between residues 20 and 29 [hIAPP(20–29) is SNNFGAILSS and rIAPP(20–29) is SNNLGPVLPP] [15–17]. Moreover, the rodent IAPP contains within this region 3 proline residues, which are absent in the human sequence. The proline residue is known to strongly unfavor *β*-sheet structures. Therefore, the hIAPP(20–29) has been suggested to be responsible for the amyloidogenic propensities of full-length hIAPP.

The decapeptide sequence of hIAPP(20–29) was shown to be able to aggregate into amyloids, whereas the corresponding rIAPP(20–29) did not [15–17]. Furthermore, shorter peptide fragments derived from this region, the penta- and hexapeptides sequences hIAPP(23–27) (FGAIL) and hIAPP(22–27) (NFGAIL), were found to be sufficient for the formation of amyloid-like structures [18]. Hence, the hIAPP(20–29) has been used as a model to study intermolecular interactions and *β*-sheet formation and was considered to be the only recognition region of IAPP.

In 2001, Fraser et al. identified a previously unrecognized amyloidogenic domain of IAPP located within residues 8–20. Synthetic peptides corresponding to this region assembled into fibrils with typical amyloid-like morphology [19]. Thereafter, we identified, using an unbiased peptide array analysis, a domain comprising the same region that showed even higher affinity recognition to IAPP as compared to the hIAPP(20–29) region [20]. IAPP was incubated with a SPOT membrane containing consecutive overlapping sequences of the full-length hIAPP. Indeed, IAPP was found to bind to the 20–29 region, however a substantially stronger interaction to a region within residues 11–20 was found. Moreover, peptide fragments within this region were shown to readily form amyloid-like structures, some of which are as short as pentapeptides, corresponding to hIAPP(14–18) and hIAPP(15–19). Thus, we suggested that this new identified region plays a central role in the recognition as well as the self-assembly of IAPP amyloid formation process.

During the last few years, accumulating data has supported our previous assumption, both directly and indirectly. However, the hIAPP(20–29) has still remained the major studied amyloidogenic region. Here, we pinpoint these supporting evidences from the recent literature in order to emphasize the relevance of the new recognition and self-assembling region. Furthermore, we provide new proofs for our hypothesis by investigating and comparing the amyloidogenic propensity of the two regions derived from the amyloidogenic cat IAPP (cIAPP).

## 2. MATERIALS AND METHODS

### 2.1. Peptide solutions

Peptides were purchased from Peptron, Inc. (Taejeon, Korea). Lyophilized peptides were dissolved in dimethyl sulfoxide (DMSO) at a concentration of 100 mM. To avoid any preaggregation, fresh stock solutions were prepared for each experiment. Peptide stock solutions were diluted into 10 mM Tris buffer, pH 7.2 to a final concentration of 2 or 5 mM and 2% or 5% DMSO, respectively.

### 2.2. Congo Red staining and birefringence

A 10 *μ*L suspension of 5 mM peptide solution aged for 1 day was allowed to dry overnight on a glass microscope slide. Staining was performed by the addition of a 10 *μ*L suspension of saturated Congo Red (CR) and NaCl in 80% ethanol (v/v) solution. Birefringence was determined with an SZX-12 stereoscope (Olympus, Hamburg, Germany) equipped with cross polarizers.

### 2.3. Transmission electron microscopy

A 10 *μ*L sample of 2 or 5 mM peptide solution aged for 1 to 3 days was placed on a 400-mesh copper grid covered by carbon-stabilized formvar film (SPI supplies, West Chester PA). After 1 minute, excess fluid was removed, and the grid was then negatively stained with 2% uranyl acetate in water for another 2 minutes. Samples were viewed in a JEOL 1200EX electron microscope operating at 80 kV.

### 2.4. Fourier-transform infrared spectroscopy

A 30 *μ*L sample of 2 mM peptide solution aged for 2 days was suspended on a polytetrafluoroethylene (PTFE) card and dried by vacuum. Peptide deposit was resuspended with D_2_O and subsequently dried. The resuspension procedure was repeated twice to ensure maximal hydrogen to deuterium exchange. Infrared spectra were recorded using a Nicolet Nexus 470 FT-IR spectrometer with a DTGS detector.

## 3. RESULTS AND DISCUSSION

### 3.1. Identification of other amyloidogenic regions within IAPP

The hIAPP(20–29) region was considered to be the central amyloidogenic domain within hIAPP. In 1999 and 2001, two other amyloidogenic regions, hIAPP(30–37) and hIAPP(8–20), were identified to be able to form amyloid-like fibrils in aqueous medium as well [19, 21]. Sequence examination of these regions reveals a single amino acid variation between the rodent and human sequences at position 18, displaying Arg or His, respectively. At the C-terminus part, rIAPP(30–37) and hIAPP(30–37) are completely homologous. Thus, it was suggested that in the rodent molecule, two potentially amyloidogenic domains exist but are separated by the proline rich domain, residues 20–29, which prevent *β*-sheet formation [4].

Shortly after the publication of our results regarding the identification of very short amyloid-forming peptides within the IAPP(14–19) region [20], another study has shown the presence of short amyloidogenic peptides within the 8–20 region [22]. By scanning a series of overlapping peptides from the 8–20 region, two peptide fragments, IAPP(12–17) and IAPP(15–20), were found to form amyloid-like structures. This study gave a strong independent support to our findings. All together these findings provided clear evidence of the presence of more than one amyloidogenic domain within IAPP.

### 3.2. Contribution of individual residues to amyloid formation by full-length IAPP

The contribution of specific residues from different regions to hIAPP fibrillization was also investigated in the context of full-length IAPP by performing single and multiple amino acid substitutions and assessing their influence on amyloid fibril formation. The ability of rIAPP variants of single-residue substitutions with amino acids from the corresponding positions of hIAPP was studied [23]. A single substitution of Arg to His at position 18 (R18H), in the full-length rIAPP, was found sufficient to render its competence for fibril formation at a small yield. Similar results were observed with the single substitutions L23F and V26I. In addition, the combination of two or three of these substitutions generally increased the ability to produce fibrils. These results show that the presence of the three proline residues in the rIAPP(20–29) domain is insufficient to abolish the ability to form fibrils. Moreover, the ability of the R18H variant of rIAPP to self-associate into amyloid fibrils suggests that other domains of IAPP except the 20–29 are involved in the self-recognition process.

The role of the histidine at position 18 in amyloid formation was further examined by assessing hIAPP fibrillization at various pH ranges [24]. The ionization state of His-18 was found to substantially affect the rate of assembly as well as the morphology of the amyloid fibrils formed by hIAPP. The aggregation process was faster at high pH (8.8), when the histidine is deprotonated, than at low pH (4.0). This fact may be physiologically relevant as mature hIAPP is stored in the *β*-cell granules at a pH of 5.5 and released into the extracellular matrix where the pH is of 7.4. Thus, the low pH in the pancreatic granules may protect hIAPP from aggregation.

Abedini and Raleigh, who performed the above-described study, further questioned the exclusive importance of the 20–29 region by designing a variant of the amyloidogenic hIAPP(8–37), containing three proline residues outside this region, at positions 17, 19, and 30 [25]. The 3×P variant had dramatically greater solubility and reduced tendency to form *β*-sheet structures compared to the wild-type polypeptide, as assayed by a variety of amyloid-detecting techniques. The authors concluded that models of IAPP fibrillization must take into account contributions of other regions within IAPP.

### 3.3. Inhibition of hIAPP amyloid formation

Based on our identification of the hIAPP(13–18) recognition site, we designed peptide inhibitors against IAPP fibrillization, which were targeted to this region. Using the previously exemplified method of incorporating *β*-breaker elements into amyloidogenic core peptides [26], we introduced a new inhibition strategy using the *α*-aminoisobutyric acid (Aib), a *β*-breaker element with extreme structural constrains [27]. Peptide fragments corresponding to the recognition domain were modified with Aib. The modified peptides completely lost their amyloidogenic potential. Furthermore, the Aib-modified peptide showed a powerful inhibitory effect on the formation of amyloid fibrils by the full-length hIAPP.

Evidently, the 20–29 region was also used as a template for inhibitor design and served as a target for inhibition. Kapurniotu et al. designed a nonamyloidogenic and bioactive mimic of hIAPP, which contains a double N-methylation of full-length hIAPP at positions G24 and I26, termed as IAPP-GI [28]. The presence of two N-methyl rests on the same side of a *β*-strand interferes with the interstrand amide hydrogen-bonding necessary for *β*-sheet formation [29]. The IAPP-GI analogue was shown to be a nanomolar affinity inhibitor of hIAPP fibrillization and cytotoxicity. However, although IAPP-GI did not form amyloid fibrils, it was found to have a “pronounced self-association propensity” and to form spheroids of up to 100 nm in diameter. Moreover, the Far-UV circular dichroism (CD) analysis of IAPP-GI indicated the presence of *β*-sheet and/or *β*-turn conformations. These insinuate the presence of another recognition and self-assembly domain besides the 20–29 region that enabled the formation of the observed structures. A reminiscent scenario exists in the study of peptide nanotubes and nanostructures, which was initiated from the search for a minimal amyloidogenic core domain of Alzheimer's *β*-amyloid polypeptide. While the Phe-Phe dipeptide was shown to form well-discrete peptide nanotubes [30], two other peptides, Cys-Phe-Phe and diphenylglycine, which are very similar to the diphenylalanine, formed closed-cage nanospheres [31]. We speculate that the remarkable inhibition ability of the IAPP-GI analogue is actually due to its high affinity recognition to the 3–18 site, as it is kept unmodified and thus provides maximal compatibility.

The search for inhibitors against amyloid formation led to the identification of insulin as an exceptional inhibitor of IAPP fibrillization [32–34]. This finding may be of high physiological importance as IAPP and insulin are costored and cosecreted from the pancreatic *β*-cell granules. Consequently, insulin is considered to form a complex with IAPP which stabilizes it and prevents it from aggregation within the granules [35, 36]. In order to reveal the molecular mechanism underlying the interaction between IAPP and insulin, we performed a molecular mapping of the interaction interface. Using a reductionist approach and peptide arrays, we located the cross-recognition sites within both polypeptides [37]. Interestingly, insulin was found to bind to the 13–18 region within IAPP. In addition, the identified recognition site within insulin, which resides within the insulin B chain, was previously shown to have sequence similarity to the hIAPP(13–18) region [35]. These findings reveal a typical amyloid inhibition mechanism for insulin and reinforce the central role of the 13–18 region: the binding of insulin to IAPP self-recognition site is mediated through sequence similarity, it interferes with IAPP self-association and prevents it from amyloid formation.

### 3.4. Interaction of IAPP with the membrane

As in the case of other amyloid-related disease, the mechanism by which IAPP causes cell destruction is assumed to involve interaction with the cell membrane. Different studies have investigated the possible interaction of IAPP with the membrane, mostly by the use of membrane mimetics such as liposomes and phospholipid assemblies. In a recent study, hIAPP was claimed to insert into phospholipid monolayer as a monomer [38]. Interestingly, it was suggested that the N-terminus of hIAPP is largely responsible for the insertion. Experiments using fragments of hIAPP showed that a peptide consisting of the 19 N-terminal residues of hIAPP efficiently inserts into a phospholipids monolayer, whereas the 20–29 residue peptide inserts much less efficiently. These results offer a possible role for the N-terminal region also in the sense of interaction with the pancreatic *β*-cells which may be part of the mechanism underlying type II diabetes pathogenesis.

### 3.5. Comparison between the two amyloidogenic regions derived from the cat IAPP

Cats are among the few mammalian species that are known to suffer from type II diabetes and from accumulation of IAPP islet amyloid deposits [39, 40]. The cat IAPP (cIAPP) sequence differs from that of hIAPP in only four amino acids, at positions 17, 18, 23, and 29. The decapeptide corresponding to cIAPP(20–29) was previously shown to be able to aggregate into amyloidal structures as well [16, 41, 42], thus supporting the apparent fibrillization role of the 20–29 region. However, both the dog and cat IAPP are identical over the 20–29 region [43] and dogs are not known to develop type II diabetes.

In order to re-evaluate the role of the two identified recognition sites of IAPP in the fibrillization process, we tested the self-assembly propensity of peptide fragments derived from the cat IAPP, cIAPP(22–29), and cIAPP(14–20). We also tested the aggregation of the corresponding human sequence peptides for reference. The peptide sequences are presented in Table 1. The peptides were dissolved into aqueous solution, under the exact same conditions, incubated for one to three days and tested for amyloid formation by electron microscopy (EM) and Congo Red (CR) birefringence. Their secondary conformation was also evaluated by Fourier-transform infrared spectroscopy (FTIR).

**Figure 1:**
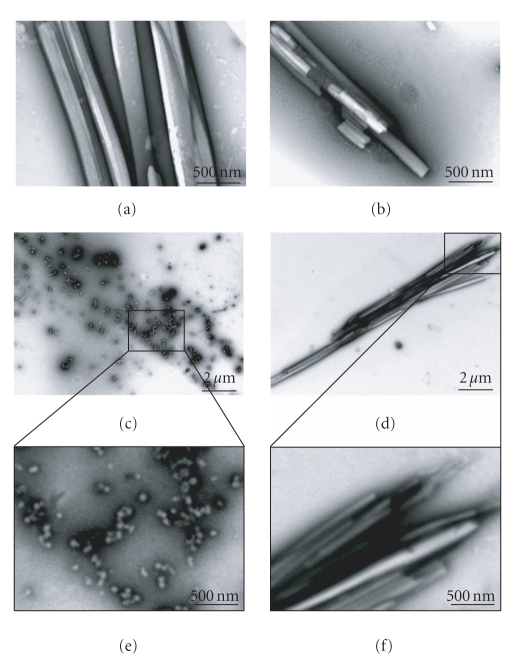
Morphology of the formed structures. Electron microscopic examination of negatively stained samples of the studied peptides: (a) C2, (b) H2, (c) C1, and (d) H1. The C1 peptide failed to form tubular structures as did the other peptides. (e) and (f) are high magnification micrographs of the marked rectangle areas of C1 and H1, respectively.

Differences between the peptides appeared already before applying the amyloid tests. Upon dilution of the peptides into aqueous solution, the H1 peptide immediately precipitated into eye-visible aggregates. The other peptide solutions appeared clear to the unarmed-eye. Examination of the peptide samples under the electron microscope revealed the presence of well-ordered tubular structures for all the peptides except the C1 peptide (Figure 1). Apparently, the peptides were found to form nanotubes rather than fibrils. The formation of tubular structures by H2 and H1 was previously shown [18, 20, 44]. Here we found that the C2 peptide can also form highly defined tubular structures. Thin fibrils were also detected in proximity with the nanotubes or in independent bundles (not shown). The C2 sample contained mostly nanotubes, the H2 sample contained nanotubes and fibrils, and the H1 sample contained also large aggregates. The C1 peptide however did not form fibrils or tubes, only less ordered spherical structures and aggregates were observed. The spherical structures may be reminiscing the ones observed for the IAPP analogue IAPP-GI [28], which were discussed above, however they are less ordered than the last ones.

**Figure 2:**
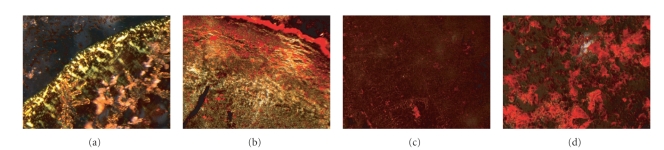
Congo Red binding and birefringence. Microscopic examination under cross-polerizers upon staining with Congo Red of samples of the studied peptides: (a) C2, (b) H2, (c) C1, and (d) H1. The C2 and H2 peptides exhibited green birefringence characteristic of amyloidal structures.

**Figure 3:**
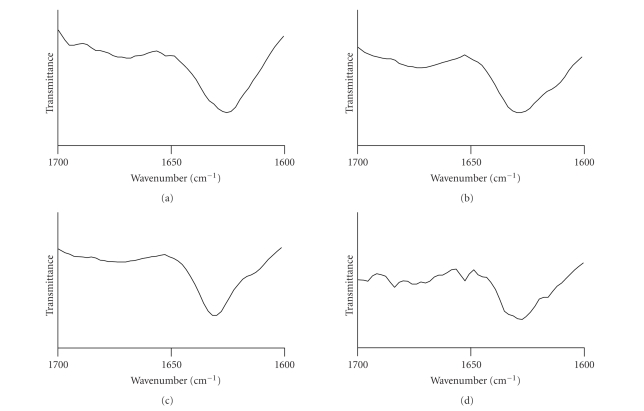
Secondary conformation of the formed structures. Fourier-transform infrared (FTIR) spectroscopy analysis of the secondary conformation of the peptides: (a) C2, (b) H2, (c) C1, and (d) H1. All the peptides exhibited spectra typical for *β*-sheet secondary structure.

Upon staining with the Congo Red dye, the C2 and H2 samples exhibited clear gold-green birefringence (Figure 2). In the H1 sample, the dye appears to be bound, however, no green color was detected. Possibly, the large amount of aggregates, which were present in addition to the fibrillar and tubular structures, were shielding over them. No birefringence or CR binding was shown in the C1 sample, the slide looked quite clean. Secondary structure analysis of the peptides by FTIR showed large *β*-sheet content for all the peptides (Figure 3). The *β*-sheet conformation is characteristic of amyloid-like structures, but is not exclusive for them. The C1 peptide, negatively detected for amyloid structures, also displayed *β*-sheet conformation which may be attributed to the spheroid structures observed by the EM or to its soluble form.

The comparison between the two cat derived peptides reveals that the C2 peptide, containing residues 14–20, is much more competent for self-assembly into amyloid-like structures than the C1 peptide, composed of residues 22–29. Although a longer version of the C1 peptide, composed of residues 20–29, was previously shown to form fibrillar structures [41, 42], in the present experimental setup it was unable to form such fibrils. We consider this diversity to result from the length of the peptide and different experimental conditions, which are in the present assay of more physiologically-relevant environment and of lower concentration. Nevertheless, when compared to the C2 peptide, C1 is dramatically less potent for fibril formation. While the C1 peptide may contain self-association properties as observed by the presence of spherical structures and *β*-sheet content, the C2 peptide exhibited self-assembly potency into well-ordered macromolecular fibrils, which were not displayed by the C1 peptide. This observation provides fresh supporting evidence for the central role of the 13–18 region within the IAPP molecule in amyloid formation: while in hIAPP both regions are highly amyloidogenic, in the cat IAPP, there is a clear difference between the two peptides.

Different behavior was also observed for the two human derived peptides H1 and H2, which may insinuate their diverse roles in the amyloidogenic process. The H1 peptide seems to be highly aggregative since visible precipitates were observed upon dilution into aqueous solution as well as under the EM. The H2 peptide however seems to have higher ability to form well-ordered structures which requires specific recognition. Its solution was visibly clear and EM examination revealed only fibrilar and tubular structures without aggregates. In addition, green birefringence upon CR binding, which is characteristic of ordered structures, was observed in the H2 sample but not in the H1 sample. Consistent with this observation is the different recognition affinity of the two peptide fragments to the full-length IAPP as we have previously showed by the peptide array assay [20].

The C1 peptide is not as aggregative as the H1 peptide, although it contains some structural features as observed by the FTIR and EM analyses. A comparison between these two sequences in their longer versions of residues 20–29 was previously performed by Ashburn and Lansbury [42]. In this study, both peptides were found to form amyloid fibrils, however, with remarkably different kinetics. The cIAPP(20–29) peptide formed fibrils approximately 13-fold more slowly than its human version. This correlates with our results regarding the different potency for fibrillization of the two sequences. It is likely that with a longer incubation period, the cIAPP(22–29) would also fibrillate eventually.

The cIAPP sequence differs from the hIAPP sequence by a Leu instead of a Phe at position 23 and a Pro instead of a Ser at position 29. Both modifications were shown to separately influence the kinetics of the peptide fibrillization [42]. It seems obvious that a Ser to Pro substitution will decrease the fibrillization potency, since Pro is a strong *β*-breaker element. Regarding the Phe to Leu substitution, we speculate that the Phe residue facilitates the fibrillization process through aromatic interactions. According to our established hypothesis, aromatic residues and interactions largely enhance the process of amyloid formation by providing directionality and stability to the growing fibril [45–49].

### 3.6. Intra-*β*-sheet mechanism for IAPP amyloid formation

The accumulated data presented in this paper prove that none of the two amyloidogenic regions within IAPP can be ignored. A possible mechanism underlying the self-assembly of IAPP into amyloid fibrils includes a role for both regions. It was already suggested in 2001 by Jaikaran and Clark that the structural transition of IAPP into amyloid fibrils involves in interactions between *β*-stranded regions within the monomer [4]. According to their model, an intramolecular *β*-sheet is formed by three *β*-strands composed of the segments 8–20, 24–29, and 32–37. Earlier secondary structure prediction based on IAPP sequence provided support to their model by predicting *β*-turns at positions 20 and 31, as well as *β*-strands for residues 14–18 and 26–29 [50]. More recently, another model for the structure of IAPP within the amyloid fibril was proposed. Accordingly, the same three *β*-strands are present, however, arranged in a zigzag planar s-shaped *β*-sheet, instead of the previously proposed e-shaped structure [51].

The presence of an intramolecular *β*-sheet in the fibrillar structure of IAPP is supported by the identification of long-range interactions between distinct residues within the IAPP molecule. Using fluorescence resonance energy transfer (FRET), the tyrosine at position 37 was shown to be close in space to the two phenylalanine residues (F23 and F15) at the fibrillogenic state of hIAPP [52]. Another interesting point arises from the genetic background of type II diabetes. Although the disease is predominantly sporadic, a mutation in hIAPP sequence, the S20G mutation, which is identified among the Japanese population, was found to be related to early onset of the disease [53]. The S20G mutant was also shown to exhibit increased fibrillization and cytotoxicity compared to wild-type hIAPP [54]. Indeed, according to the intra-*β*-sheet models, the mutation is placed in a *β*-turn region, which is favorable for the glycine residue.

## 4. CONCLUSIONS

The formation of amyloid deposits by IAPP appears to play a central role in the pathogenesis of type II diabetes. Knowledge regarding the exact mechanism of this process may be highly relevant for the development of therapeutic agents for the treatment of the disease. Although it is clear that many environmental factors are involved in this process, understanding the amyloid formation route at the molecular level may be highly valued. In this paper, we have summarized results from several research groups which support the notion that the 20–29 region within IAPP cannot be considered as the sole amyloidogenic core of the polypeptide. By reviewing previously published data, by us and others, as well as providing experimental data regarding the amyloidogenicity of the 13–18 region derived from the cat IAPP, we emphasized the essential role of the 13–18 region in the fibrillization process of IAPP. We conclude that an intramolecular *β*-sheet structure, formed by at least the two discussed regions, may provide a very good mechanism to the discussed observations.

## Figures and Tables

**Table 1:** The studied peptides. Peptide fragments corresponding to regions within the human and cat IAPP were tested for the ability to form amyloid-like structures.

Name	Origin	Sequence	Residues
C1	cat	NLGAILSP	22–29
H1	human	NFGAILSS	22–29
C2	cat	NFLIRSS	14–20
H2	human	NFLVHSS	14–20
